# Consensus Comparative Analysis of Human Embryonic Stem Cell-Derived Cardiomyocytes

**DOI:** 10.1371/journal.pone.0125442

**Published:** 2015-05-04

**Authors:** Shaohong Zhang, Ellen Poon, Dongqing Xie, Kenneth R. Boheler, Ronald A. Li, Hau-San Wong

**Affiliations:** 1 Department of Computer Science, Guangzhou University, Guangzhou, P.R. China; 2 Stem Cell & Regenerative Medicine Consortium, University of Hong Kong, Hong Kong; 3 Department of Computer Science, City University of Hong Kong, Hong Kong; University of Kansas Medical Center, UNITED STATES

## Abstract

Global transcriptional analyses have been performed with human embryonic stem cells (hESC) derived cardiomyocytes (CMs) to identify molecules and pathways important for human CM differentiation, but variations in culture and profiling conditions have led to greatly divergent results among different studies. Consensus investigation to identify genes and gene sets enriched in multiple studies is important for revealing differential gene expression intrinsic to human CM differentiation independent of the above variables, but reliable methods of conducting such comparison are lacking. We examined differential gene expression between hESC and hESC-CMs from multiple microarray studies. For single gene analysis, we identified genes that were expressed at increased levels in hESC-CMs in seven datasets and which have not been previously highlighted. For gene set analysis, we developed a new algorithm, consensus comparative analysis (CSSCMP), capable of evaluating enrichment of gene sets from heterogeneous data sources. Based on both theoretical analysis and experimental validation, CSSCMP is more efficient and less susceptible to experimental variations than traditional methods. We applied CSSCMP to hESC-CM microarray data and revealed novel gene set enrichment (e.g., glucocorticoid stimulus), and also identified genes that might mediate this response. Our results provide important molecular information intrinsic to hESC-CM differentiation. Data and Matlab codes can be downloaded from S1 Data.

## Introduction

Human embryonic stem cells (hESC) self-renew; their differentiation to the cardiac lineage represents a potentially unlimited source of cardiomyocytes (CMs) for therapies and as experimental models to investigate mechanisms involved in human cardiac development and for disease progression. A genome-wide characterization of the molecular phenotype of hESC-CMs is important for these applications. Microarray experiments have been performed by various groups and have shown that hESC-CMs expressed contractile genes, transcription factors, potassium channels and Ca handling genes that are commonly found in the heart [1–7]. In spite of this, there is remarkable divergence among these studies. [4] evaluated their list of 1311 genes upregulated in CM with those presented by [1], and by [2] and showed that only 33 genes were shared by all three studies. This divergence may be attributed to a number of experimental variables such as hESC strains, differentiation conditions, culture duration and microarray platform/thresholds used (Table 1). For instance, [3] and [2] generated CMs by non-directed, spontaneous embryoid body formation without addition of growth factors, while [7] performed stage-specific addition of growth factors including bFGF, BMP4, activin etc. to direct cardiac differentiation. In addition, 4 different hESC lines were used in the 6 studies; it has been shown that different hESC lines have predetermined preferences to become ventricular, atrial and pacemaker CMs with different electrophysiological properties [8]. It is to be expected that the variables described above would impact the transcriptome of CMs generated. A consensus comparative analysis from multiple studies would thus be invaluable to distinguish between factors/pathways crucial for CM differentiation and those that are reflections of experimental conditions.

**Table 1:** Summary of related studies and filtered differential expression conditions.

Study	Strain	Differentiation Protocol	Age	Isolation	% Yield	Method and Parameter	#Sig. Gene
[1]	HES2	END2 co-culture, no FBS	12d	Mechanical dissection	Uncertain	p < 0.01; FC≥2	2608
[2]	SA002	Spontaneous EB formation	< 22d	Mechanical dissection	Uncertain	SAM, FDR < 0.04;FC≥5	530
[3]	H9	Spontaneous EB formation	14d	Percoll gradient	40–45%	FC≥2	5504
[4]	HES3	CM differentiation with transgenic line	21d	Antibiotic selection	> 99%	FDR < 0.05; FC≥2	1311
[5]	SA002	Spontaneous EB formation	21d	Mechanical dissection	Uncertain	FC≥2	1781
[5]	SA002	Spontaneous EB formation	49d	Mechanical dissection	Uncertain	FC≥2	1883
[7]	HES2	Directed differentiation with growth factor	21d	Transduction and sorting	> 99%	FC≥2	2896

Gene set analysis is more effective than single gene analysis in identifying consensus expression patterns across different data sets in general, and represents a recent and successful analysis tool family commonly adopted in bioinformatics studies [9–12]. These tools usually adopt a complete data matrix or a large ordered gene list as the input, and assess statistical significance based on multiple random permutations. While some groups have made their data matrices publicly available, most microarray papers involving hESC-CMs only provide lists of differentially expressed genes [1, 2, 4]. It is therefore difficult to perform gene set comparison across multiple studies using heterogeneous data sources (including both data matrices and lists of differentially expressed genes). In view of these challenges, we devised a novel algorithm to identify gene sets that are enriched in hESC-CMs relative to hESC in multiple studies. We showed that our new algorithm has improved properties compared to traditional methods, and we identified differential expression changes in gene sets that have not been previously reported.

### Contribution of this paper

We are the first to perform consensus comparative investigation of hESC-CMs to identify genes/gene sets upregulated in hESC-CMs independent of experimental conditions. We have identified novel enrichment of genes and gene sets in hESC-CMs, and our results provide valuable information about the molecular program that is active in hESC-CMs. The main computational contribution of our work is the proposal of a new gene set analysis method, i.e., consensus comparative analysis (CSSCMP), to identify commonly enriched gene sets across multiple studies based on lists of differentially expressed genes (without data matrices). From both theoretical analysis and experimental validation, we show that our CSSCMP method has a number of desirable properties: (a) Capability to detect randomness in the input. (b) Improvement of efficiency through relaxing the condition of using a large number of random permutations commonly adopted by traditional gene set based analysis methods [9]. (c) Mitigation of the problem of gene set size dependence. (d) Integration of information from multiple heterogeneous data sources for improved analysis.

## Related work

Transcriptomic profiling studies have been performed to characterize hESC-CMs and to identify gene regulatory mechanisms that control the differentiation of hESCs into CMs [1–6]. [1] assessed time-dependent gene expression patterns of hESCs differentiating towards CMs. [2] then identified genes and pathways that were upregulated in hESC-CM clusters compared to undifferentiated hESCs. [3] and [4] used CMs of higher purities (30–40%, > 99% respectively) to compare the transcriptome of CMs with hESC and fetal heart cells, while [7] compared ventricular hESC-CMs with fetal and adult CMs of the same lineage. A later study by [5] focused on the expression of ion channel and *Ca* ^2+^-handling genes in hESC-CM clusters. Most of the hESC-CM studies only provided lists of significantly differentially expressed genes [1, 2, 4], rather than the complete gene expression datasets. [4] and Synnergren et al. examined genes commonly upregulated in 2–4 studies, but gene set analysis has not been performed [13].

Gene set analysis methods are more effective in the search for consensus results than single gene analysis methods [9–12]. These tools can roughly be divided into two categories: (1) microarray data based methods, which in general access the full data matrices. Representative examples include GSEA [9], SAFE [14], SAM-GS [15];(2) significant gene list based methods, which utilize lists of significantly differentially expressed genes as input. Representative examples include DAVID [10], FuncAssociate [16], WebGestalt [17] and Bingo [18]. However, to our best knowledge, there are no effective tools that can identify differentially expressed gene sets from heterogeneous data sources which include a combination of full data matrices and gene lists with different thresholds for fold changes (FC).

## Materials and Methods

### Materials

We collected data from microarray studies [1–5, 7] as shown in Table 1. The first six data sets correspond to heterogeneous CM populations with different purity levels, while the last one [7] consists of purified hESC-CMs of the ventricular lineage. In view of the diverse nature of the data sets, our main focus is on non-lineage specific analysis of the gene expression patterns of hESC-CMs, instead of those associated with any particular chamber-specific lineage. For several studies [1, 2, 4], only lists of differentially expressed genes were available, and the corresponding methods and parameters adopted by the original authors are shown in the column ‘Method and Parameter’ in Table 1. For the other studies, FC thresholds were set to 2 in order to provide a uniform basis for comparison. We extracted two related gene set collections, named the general Homo sapiens gene sets on Biological Process (HSBP) and the subset from the work of British Heart Foundation-University College London on Biological Process (UCLBP), respectively, from the Gene Ontology and Gene Ontology Annotation databases, in the well-known.gmt file format. HSBP groups genes based on general biological processes and is most suitable for examining gene functions. UCLBP is composed of genes mainly related to heart development. We used version 1.1.2681 of the file gene ontology.1.0.obo (Time stamp: 06:03:2012 19:30, downloaded from the GO official website at http://www.geneontology.org/ontology/oboformat_1_0/gene ontology.1_0.obo). For the Homo Sapiens annotation file, we used version 1.225 of the file gene_association.goa human (Time stamp: 06:03:2012, downloaded from the Gene Ontology Annotation (UniProt-GOA) Database at ftp://ftp.ebi.ac.uk/pub/databases/GO/goa/HUMAN/gene association.goa human.gz). The UCLBP gene set collection is constructed based on the work of British Heart Foundation-University College London (BHF-UCL) GO teams and their coworkers on the representation of heart development in GO [19], which is accessed from the file ftp://ftp.ebi.ac.uk/pub/databases/GO/GOA/bhf-ucl/gene_association.goa_bhf-ucl.gz. To use up-to-date gene ontology and annotation data, we construct these two gene set collections using a similar method as is adopted for the GSEA official MsigDB C5 gene sets (see http://www.broadinstitute.org/gsea/msigdb/collection_details.jsp#C5). Specifically, only entries associated with the following evidence codes were included: Inferred from Direct Assay (IDA), Inferred from Physical Interaction (IPI), Inferred from Mutant Phenotype (IMP), Inferred from Genetic Interaction (IGI), Inferred from Expression Pattern (IEP), Inferred from Sequence or Structural Similarity (ISS), and Traceable Author Statement (TAS). We removed gene sets with more than 500 genes or fewer than 15, to exclude very broad categories or very narrow ones, as suggested by the GSEA user guide [9]. Specifically, there are 1564 gene sets in the HSBP gene set collection and 966 ones in UCLBP. Note that the HSBP gene set collection is more general since it is related to most of the Homo Sapiens biological processes. On the other hand, the UCLBP gene set collection focuses on heart development [19] and therefore it is more specific than the former one. Through a closer inspection of the two gene set collections, we find that there are 915 gene sets in common in both gene set collections, and therefore UCLBP can roughly be viewed as a subset of HSBP.

### Methods

Given *D* individual studies, we extracted a combined gene set *ψ* ^*C*^ of all *N* ^*C*^ genes in the different studies (The superscript is used to distinguish entities associated with the combined gene set from those of a specific gene set). Specifically, we constructed a *N* ^*C*^ × *D* overall contingency matrix *M* with entries *m*_*ij*_ as follows:

$$\begin{array}{c} {\left[M\right]}_{ij}=m_{ij}=\{\begin{array}{cc} 1 & \text{if}\;\text{gene}\;i\;\text{is}\;\text{up-regulated}\;\text{in}\;\text{study}\;j,\\ 0 & \text{otherwise}. \end{array} \end{array}$$ (1)

Given a specific gene set *ψ* ^*G*^ with *N* ^*G*^ genes within the combined gene set *ψ* ^*C*^, we can extract the corresponding contingency matrix *M* ^*G*^ from the overall contingency matrix. An intuitive method is to compute the counting score (CS) of the lower triangular entries of the matrix *L* ^*G*^ = (*M* ^*G*^)^*T*^ *M* ^*G*^ (here the notation (*M* ^*G*^)^*T*^ refers to the transpose of the matrix (*M* ^*G*^)) as follows

$$\begin{array}{c} S_{cs}\left(M^{G}\right)=\sum_{a=1}^{D}\sum_{b=1}^{a}L_{ab}^{G}=\sum_{a=1}^{D}\sum_{b=1}^{a}\sum_{i=1}^{N^{G}}m_{ia}^{G}m_{ib}^{G}. \end{array}$$ (2)

The counting score reflects both the co-association of different study pairs and the number of up-regulated genes in each study. However, it is notable that it suffers from the problem of producing non-zero values for random contingency matrices, and its dependence on the matrix size, similar to problems well discussed for the Rand index [20] as a cluster validity measure [21–24]. For the *j*-th study, the estimated probability of a gene to be up-regulated based on the overall contingency matrix can be computed as

$$\begin{array}{c} {\hat{p}}_{j}=\frac{\sum_{i=1}^{N^{C}}m_{ij}}{N^{C}}. \end{array}$$ (3)

Thus the expected value of CS corresponding to a *N* ^*R*^ × *D* random contingency matrix *M* ^*R*^ can be computed as

$$\begin{array}{c} \bar{S}=𝔼\left[\sum_{a=1}^{D}\sum_{b=1}^{a}\sum_{i=1}^{N^{R}}\left(m_{ia}^{R}m_{ib}^{R}\right)\right]\approx N^{R}\sum_{a=1}^{D}\sum_{b=1}^{a}{\hat{p}}_{a}^{R}{\hat{p}}_{b}^{R}, \end{array}$$ (4)

where ${\hat{p}}_{a}^{R}$, ${\hat{p}}_{b}^{R}$ are estimated up-regulation probabilities based on the random contingency matrix. The second step follows from the approximation of the expected value based on the estimated probabilities when *N* ^*R*^ is large. We can observe from Eq (4) that the score value is not zero, and is correlated to the size of the gene set *N* ^*R*^. Motivated by the adjusted Rand index and related clustering measures proposed in [21, 23], which expresses the modified measures in the form of $\frac{S_{cs}-\bar{S}}{S_{max}-\bar{S}}$, where *S*_*cs*_ and *S*_*max*_ represent the original score and the maximum possible score respectively, and $\bar{S}$ represents the expected score value for random inputs, we propose an improved consensus comparative analysis (CSSCMP) score based on the contingency matrix. The maximum score value corresponding to the contingency matrix *M* ^*G*^ can be readily found when all the entries are ones, i.e., all the entries of the matrix (*M* ^*G*^)^*T*^ *M* ^*G*^ equal *N* ^*G*^. Thus, the maximum score value is computed as

$$\begin{array}{c} S_{max}=\sum_{a=1}^{D}\sum_{b=1}^{a}\sum_{i=1}^{N^{G}}m_{ia}^{G}m_{ib}^{G}=\frac{N^{G}D\left(D-1\right)}{2}. \end{array}$$ (5)

Therefore, the consensus comparative analysis score can be computed as

$$\begin{array}{l} S_{CSSCMP}\left(M^{G}\right)\;=\;\frac{S_{cs}-\bar{S}}{S_{max}-\bar{S}}\\ =\;\frac{\sum_{a=1}^{D}\sum_{b=1}^{a}\sum_{i=1}^{N^{G}}m_{ia}^{G}m_{ib}^{G}-N^{G}\sum_{a=1}^{D}\sum_{b=1}^{a}{\hat{p}}_{a}{\hat{p}}_{b}}{\frac{N^{G}D\left(D-1\right)}{2}-N^{G}\sum_{a=1}^{D}\sum_{b=1}^{a}{\hat{p}}_{a}{\hat{p}}_{b}}. \end{array}$$ (6)

Compared to conventional gene set analysis methods, such as GSEA [9], our proposed CSSCMP method has a number of advantages in handling imperfect data prevalent in the studies of hESC-CMs: (1) CSSCMP only uses lists of significantly differentially expressed genes, thus it is readily applicable to the analysis of multiple studies since many studies only release their significant gene lists rather than the full microarray data; (2) CSSCMP does not require performing multiple random permutation trials, which is commonly used in traditional methods. In general, these kinds of random permutation trials require significant computation time (e.g., 1000 trials are commonly used in GSEA). As a result, our method improves computational efficiency; (3) The CSSCMP score value is close to zero with random input contingency matrices, which allows our approach to distinguish meaningful inputs from trivial ones. (4) CSSCMP is less sensitive to the size of gene sets, which is also an important concern in traditional gene set analysis methods. Verifications of the last two properties are presented as follows, and confirmed in experiments using both simulated and real data.

#### Proposition: Detection of randomness

CSSCMP is close to zero for a random input contingency matrix, and is less sensitive to the size of gene sets. The verification is straightforward. For a *N* ^*R*^ × *D* random input contingency matrix *M* ^*R*^, when the number of genes in the random gene set *N* ^*R*^ is large enough, we have

$$\begin{array}{ccc} S_{csscmp}\left(M^{R}\right) & = & \frac{S_{cs}-\bar{S}}{S_{max}-\bar{S}}\\ & = & \frac{\sum_{a=1}^{D}\sum_{b=1}^{a}\sum_{i=1}^{N^{R}}\left(m_{ia}^{R}m_{ib}^{R}\right)-N^{R}\sum_{a=1}^{D}\sum_{b=1}^{a}{\hat{p}}_{a}^{R}{\hat{p}}_{b}^{R}}{\frac{N^{R}D\left(D-1\right)}{2}-N^{R}\sum_{a=1}^{D}\sum_{b=1}^{a}{\hat{p}}_{a}^{R}{\hat{p}}_{b}^{R}}\\ & \approx & \frac{\sum_{a=1}^{D}\sum_{b=1}^{a}{\hat{p}}_{a}^{R}{\hat{p}}_{b}^{R}-\sum_{a=1}^{D}\sum_{b=1}^{a}{\hat{p}}_{a}^{R}{\hat{p}}_{b}^{R}}{\frac{D(D-1)}{2}-\sum_{a=1}^{D}\sum_{b=1}^{a}{\hat{p}}_{a}^{R}{\hat{p}}_{b}^{R}}\\ & = & 0. \end{array}$$ (7)

The third step follows from the approximation of the expected value of CS based on the estimated up-regulation probabilities when *N* ^*R*^ is large. Note that the result is less sensitive to the size of gene set *N* ^*R*^, since this factor is removed in the third step.

## Results

### Gene based consensus comparative analysis in hESC-CMs relative to hESCs

**Fig 1:**
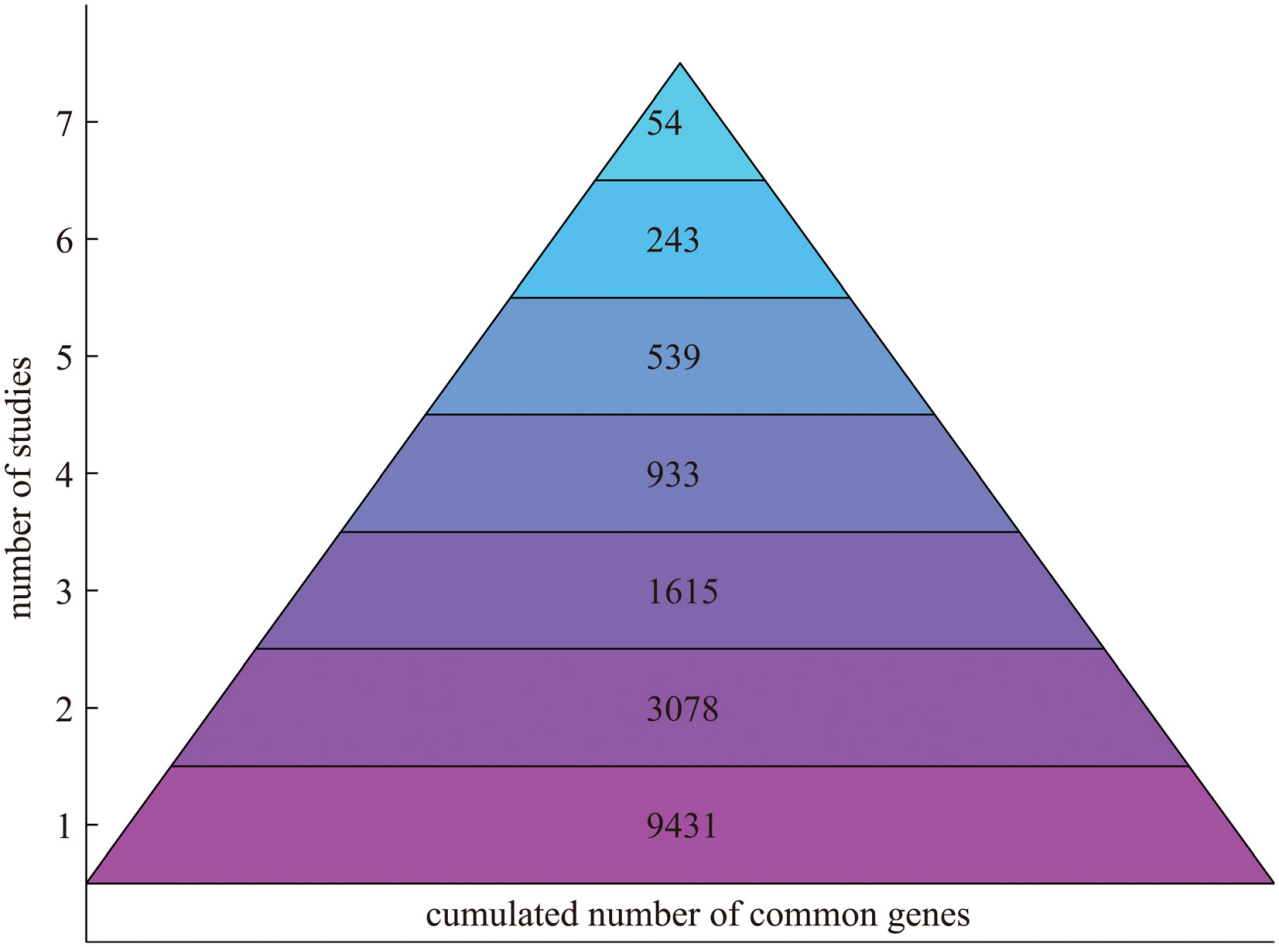
Statistics of common genes in hESC-CMs relative to hESCs in multiple studies.

We first examined genes commonly upregulated in multiple individual studies. A pyramid chart for statistics of commonly enriched genes in hESC-CMs relative to hESCs in multiple studies is shown in Fig 1. Only a small number (i.e., 53) of genes are enriched in all studies while a large number (i.e., 9431) is found in at least one study. This implies that interpretation of the results at the level of gene sets is important besides the identification of individual genes. In this section we will focus on consensus comparative analysis in hESC-CMs relative to hESCs based on individual genes. Gene set based consensus comparative analysis will be performed in the following section.

#### Genes uniformly enriched in hESC-CMs relative to hESCs in all studies

We first focused on genes that were uniformly enriched in hESC-CMs relative to hESCs, as listed in Table 2. Up-regulated genes included those known to be crucial for heart development/function such as transcription factors e.g., MEF2C and GATA4, contractile genes e.g., MYH7 and TNNC1 etc., as well as ion transport genes e.g., ATP2A2 and PLN etc. Interestingly, as shown in Table 3, 40% (22 out of 54) of our upregulated genes were not highlighted by the hESC-CM microarray studies examined.

**Table 2:** Common genes uniformly overexpressed in hESC-CMs compared to hESCs in all studies.

**GENE SYMBOL**	**ENSEMBL GENE ID**	**GENE NAME**
MEF2C	ENSG00000081189	myocyte enhancer factor 2C
APOBEC2	ENSG00000124701	apolipoprotein B mRNA editing enzyme, catalytic polypeptide-like 2
COL21A1	ENSG00000124749	collagen, type XXI, alpha 1
TNNC1	ENSG00000114854	troponin C type 1 (slow)
IL6ST	ENSG00000134352	interleukin 6 signal transducer (gp130, oncostatin M receptor)
RRAD	ENSG00000166592	Ras-related associated with diabetes
TTN	ENSG00000155657	titin
SYNPO2L	ENSG00000166317	synaptopodin 2-like
TGFB2	ENSG00000092969	transforming growth factor, beta 2
EDNRA	ENSG00000151617	endothelin receptor type A
KIFAP3	ENSG00000075945	kinesin-associated protein 3
GATA4	ENSG00000136574	GATA binding protein 4
LMOD1	ENSG00000163431	leiomodin 1 (smooth muscle)
PPP1R14C	ENSG00000198729	protein phosphatase 1, regulatory (inhibitor) subunit 14C
MB	ENSG00000198125	myoglobin
ACTA2	ENSG00000107796	actin, alpha 2, smooth muscle, aorta
MICAL2	ENSG00000133816	microtubule associated monoxygenase, calponin and LIM domain containing 2
MYH7	ENSG00000092054	myosin, heavy chain 7, cardiac muscle, beta
ACTN2	ENSG00000077522	actinin, alpha 2
MYH6	ENSG00000197616	myosin, heavy chain 6, cardiac muscle, alpha
FLNC	ENSG00000128591	filamin C, gamma (actin binding protein 280)
TNNT2	ENSG00000118194	troponin T type 2 (cardiac)
CLIC5	ENSG00000112782	chloride intracellular channel 5
HSPB7	ENSG00000173641	heat shock 27kDa protein family, member 7 (cardiovascular)
SMPX	ENSG00000091482	small muscle protein, X-linked
CTSB	ENSG00000164733	cathepsin B
HSPB3	ENSG00000169271	heat shock 27kDa protein 3
PRNP	ENSG00000171867	prion protein
NPPA	ENSG00000175206	natriuretic peptide precursor A
ZNF436	ENSG00000125945	zinc finger protein 436
MYL7	ENSG00000106631	myosin, light chain 7, regulatory
MYL4	ENSG00000198336	myosin, light chain 4, alkali; atrial, embryonic
MYL3	ENSG00000160808	myosin, light chain 3, alkali; ventricular, skeletal, slow
COL2A1	ENSG00000139219	collagen, type II, alpha 1
MEIS1	ENSG00000143995	Meis homeobox 1
MSX2	ENSG00000120149	msh homeobox 2
PPP1R3C	ENSG00000119938	protein phosphatase 1, regulatory (inhibitor) subunit 3C
MEIS2	ENSG00000134138	Meis homeobox 2
AGT	ENSG00000135744	angiotensinogen (serpin peptidase inhibitor, clade A, member 8)
FBN2	ENSG00000138829	fibrillin 2
HRC	ENSG00000130528	histidine rich calcium binding protein
TBX3	ENSG00000135111	T-box 3
CRIP2	ENSG00000182809	cysteine-rich protein 2
CSRP3	ENSG00000129170	cysteine and glycine-rich protein 3 (cardiac LIM protein)
TNNI1	ENSG00000159173	troponin I type 1 (skeletal, slow)
RASSF5	ENSG00000136653	Ras association (RalGDS/AF-6) domain family member 5
CDKN1A	ENSG00000124762	cyclin-dependent kinase inhibitor 1A (p21, Cip1)
ATP2A2	ENSG00000174437	ATPase, Ca++ transporting, cardiac muscle, slow twitch 2
PKP2	ENSG00000057294	plakophilin 2
SVIL	ENSG00000197321	supervillin
PLN	ENSG00000198523	phospholamban
RGS5	ENSG00000143248	regulator of G-protein signaling 5
BMP5	ENSG00000112175	bone morphogenetic protein 5
TMOD1	ENSG00000136842	tropomodulin 1

**Table 3:** Genes that are significantly upregulated for hESC-CMs in the seven data sets. Only genes that have not been highlighted in the individual studies are shown. Genes that are enriched by more than 10-fold in hESC-CMs relative to both hESC and hESC-derived embryoid bodies are shown in bold. Fold changes are based on data from [4], who used purified CMs and who compared hESC-CM gene expression with both hESC and hESC-derived embryoid bodies.

Genes	Name	CM/ES	CM/EB
**JPH2**	junctophilin 2	72	31
COL21A1	collagen, type XXI, alpha 1	47	2
IL6ST	interleukin 6 signal transducer (gp130, oncostatin M receptor)	161	5
KIFAP3	kinesin-associated protein 3	10	3
LMOD1	leiomodin 1 (smooth muscle)	6	4
**MICAL2**	microtubule associated monoxygenase, calponin and LIM domain containing 2	36	22
FLNC	filamin C, gamma (actin binding protein 280)	5	3
CTSB	cathepsin B	3	3
ZNF436	zinc finger protein 436	15	4
MEIS1	Meis homeobox 1	44	2
MSX2	msh homeobox 2	167	5
**PPP1R3C**	protein phosphatase 1, regulatory (inhibitor) subunit 3C	23	10
MEIS2	Meis homeobox 2	44	2
AGT	angiotensinogen (serpin peptidase inhibitor, clade A, member 8)	52	4
RASSF5	Ras association (RalGDS/AF-6) domain family member 5	31	6
CDKN1A	cyclin-dependent kinase inhibitor 1A (p21, Cip1)	3	4
PKP2	plakophilin 2	16	5
SVIL	supervillin	10	5
**RGS5**	regulator of G-protein signaling 5	95	11

These genes were commonly upregulated in hESC-CMs independent of culture condition and differentiation protocol, and are likely to be important for early human CM differentiation. Of these, 6 genes show CM-specific expression and were 10-fold enriched in hESC-CMs relative to both undifferentiated hESC and mixed embryoid bodies culture [4]. This list included transcripts which are known to be important in the heart and whose presence in hESC-CMs has not been reported. JPN2 and RGS5 are such examples [25–27], and are likely to be important for controlling calcium handling and cardiac repolarization in hESC-CMs. In addition, we identified upregulation of genes with unknown roles in the heart, and they are MICAL2 and CPNE5. Both genes are strongly upregulated in hESC-CMs e.g. MICAL2 expression is 36 and 22 fold higher in hESC-CMs compared to hESCs and embryoid bodies respectively. In addition, both genes were also enriched by more than 8 fold in human fetal and adult CMs relative to hESCs and embryoid bodies. MICAL2 is a cytoskeletal protein involved in adhesion and actin polymerization [28]. CPNE5 encodes a poorly characterized Ca binding membrane protein [29]. It is unclear what roles they play in heart development and function and merits further attention.

#### Statistics of literature-curated marker genes of important biological processes in multiple studies

**Fig 2:**
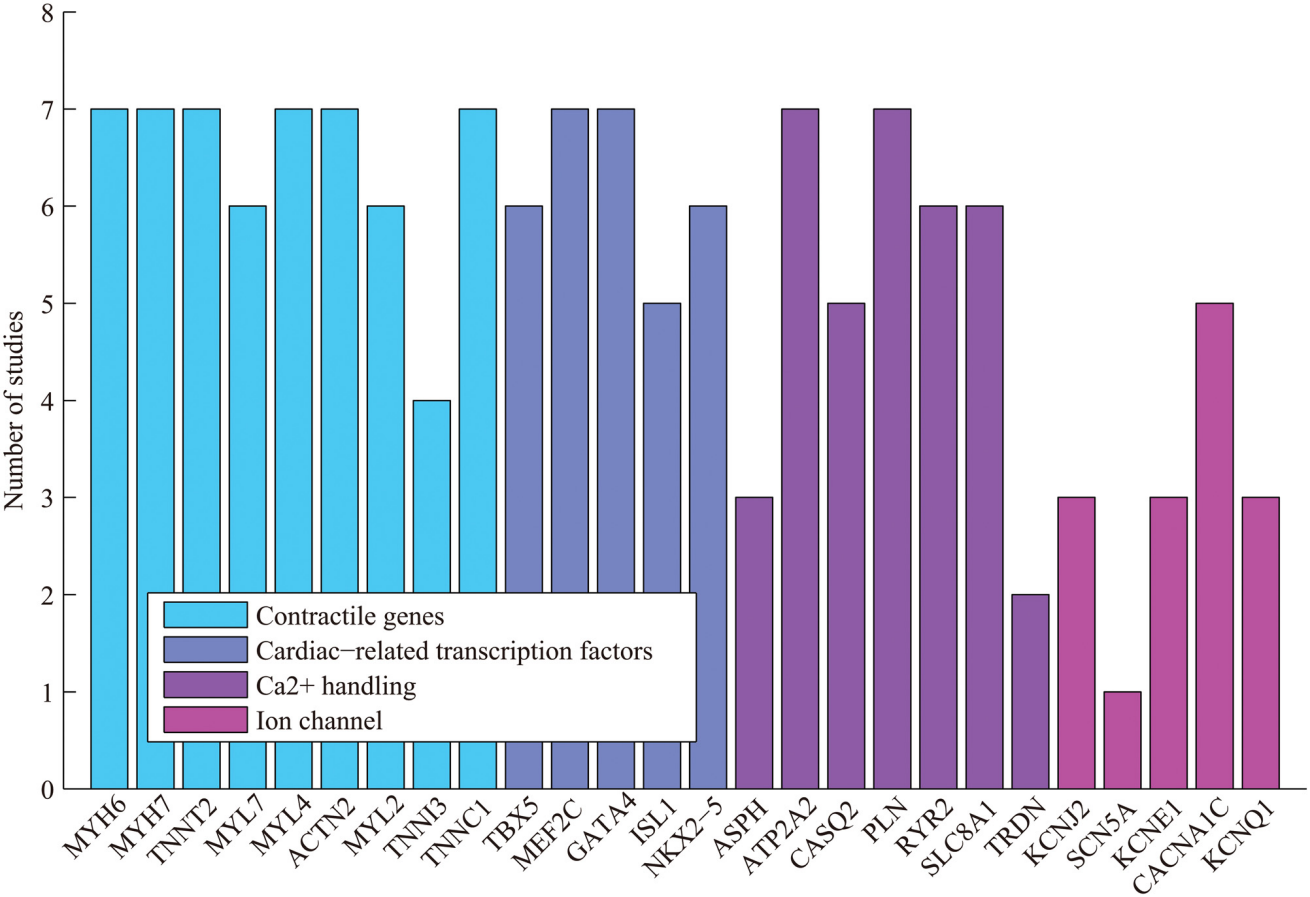
Enrichment of representative genes of biological processes closely associated with heart development and function.

We next examined the enrichment of selected genes known to be important for cardiac functions such as contractile genes, cardiac transcription factors, *Ca* ^2+^ handling genes, and ion channels, and found significant variation among individual studies, as shown in Fig 2. Gene sets associated with heart development, contraction, *Ca* ^2+^ handling were uniformly upregulated in all studies examined, but the upregulation of genes within these gene sets were not uniform. 6 of the 9 contractile genes studied were enriched in all of the studies. By contrast, none of the ion channel genes were enriched in all seven studies. For instance, KCNE1 and KCNQ1 were enriched in only 3 out of 7 studies. Thus, in order to observe the enrichment of gene groups corresponding to different biological processes and functions, gene set analysis is required as an important complementary approach besides individual gene analysis.

#### Enriched biological process categories for uniformly enriched genes in different numbers of studies

**Fig 3:**
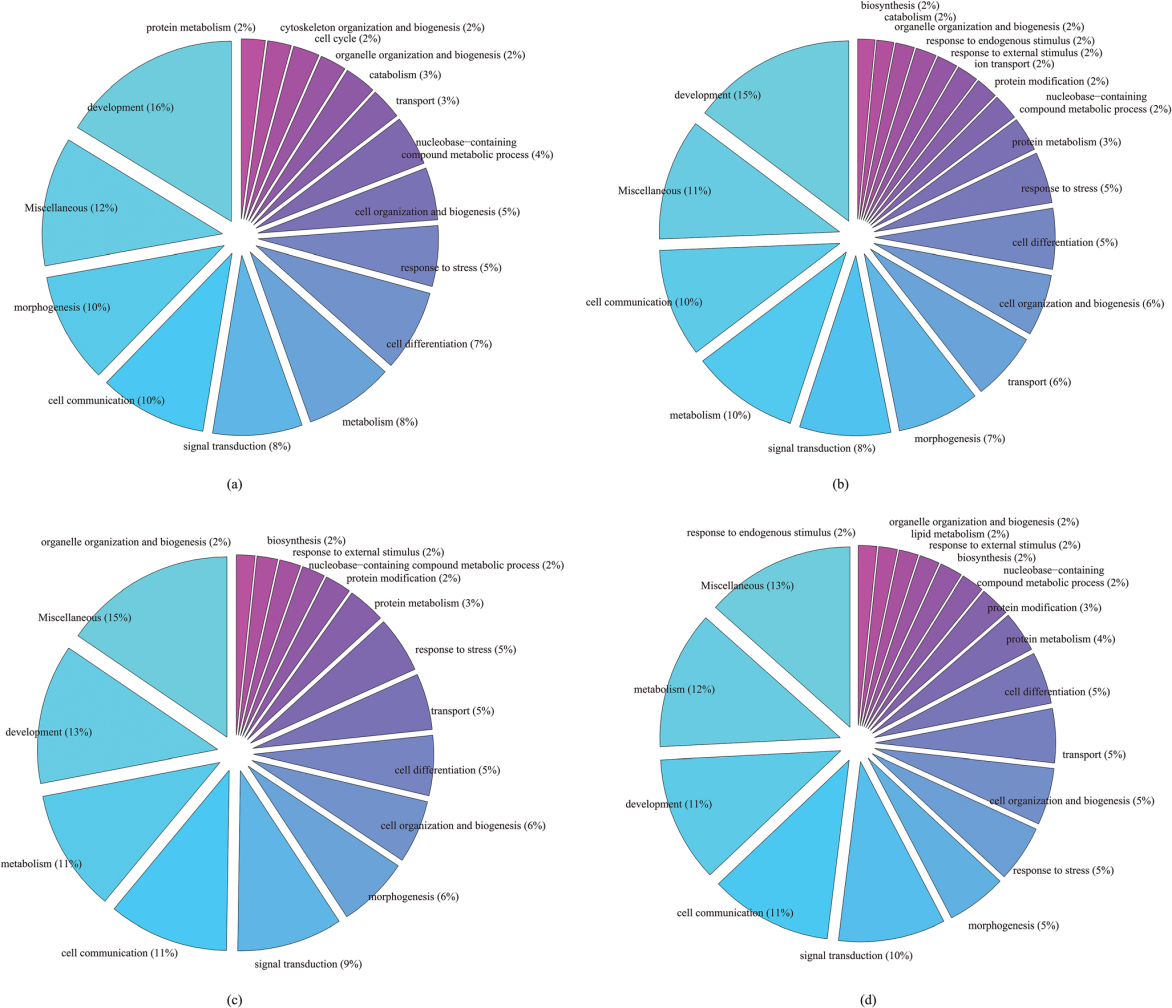
Enriched GO BP categories for uniformly enriched genes in different numbers of studies: (a) 7 studies, (b) 6 studies; (c) 5 studies; (d) 4 studies.

To further evaluate variability in gene- and gene-set based methods, we next assessed the gene ontology affiliations of genes uniformly enriched in multiple studies. Specifically, we identified genes that were significantly enriched in CMs in at least 7, 6, 5 and 4 studies respectively, and extracted their Gene Ontology annotation as shown in Fig 3. The top BP categories were development, morphogenesis, cell communication, metabolism, and signal transduction. The GO enrichment pattern was largely conserved irrespective of the number of studies used. This shows that gene set based analysis is less sensitive to variations in different studies than gene based analysis. The results also confirmed that the 7 studies were closely related.

### Gene set based consensus comparative analysis on hESC-CMs

In this section, we verified the properties of our method on both simulated data and real data. Then, we presented the results of CSSCMP on hESC-CMs.

#### Verification of the properties of consensus comparative analysis

**Fig 4:**
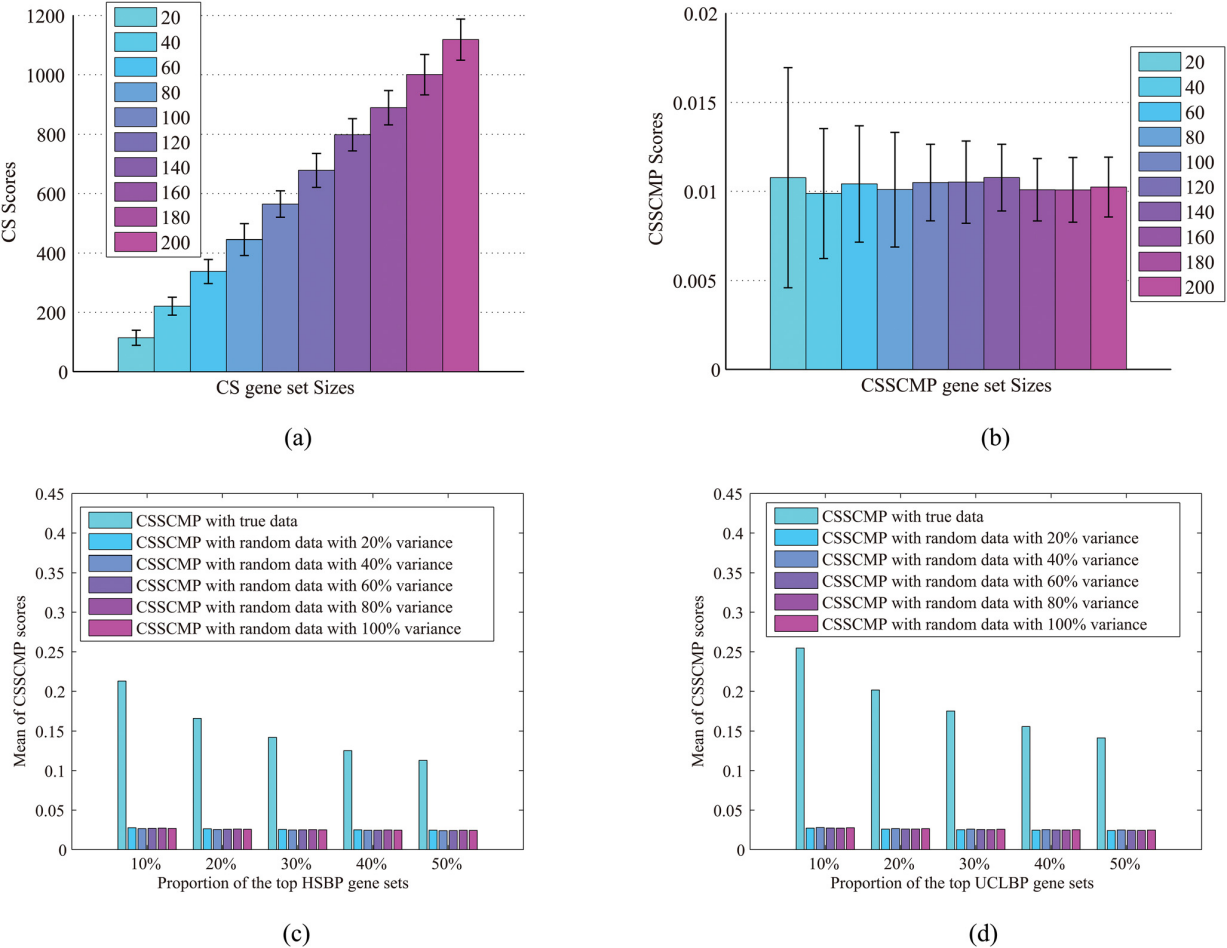
Verification of the properties of consensus comparative analysis compared with random data. (a) Plots of CS scores based on random contingency matrices of 20 individual studies with various gene set sizes; (b) Plots of CS scores based on random contingency matrices of 20 individual studies with various gene set sizes; (c) Mean of CSSCMP scores of top HSBP gene sets compared with the hESC-CM data and random data with different levels of variance; (d) Mean of CSSCMP scores of top UCLBP gene sets compared with the hESC-CM data and random data with different levels of variance. The CS score heavily depends on the gene set sizes, while CSSCMP scores are insensitive to the size of gene sets and consistently small under random data with different levels of variance.

We first investigated our CSSCMP analysis method using random contingency matrices of 20 individual studies with various gene set sizes. As shown in Fig 4A and 4B, the CS score heavily depended on gene set sizes, while CSSCMP scores were consistently small and insensitive to the sizes. These observations suggested that CSSCMP was capable of detecting randomness and was more robust against the effect of gene set sizes, both of which were in agreement with the proposition in Eq (7).

**Fig 5:**
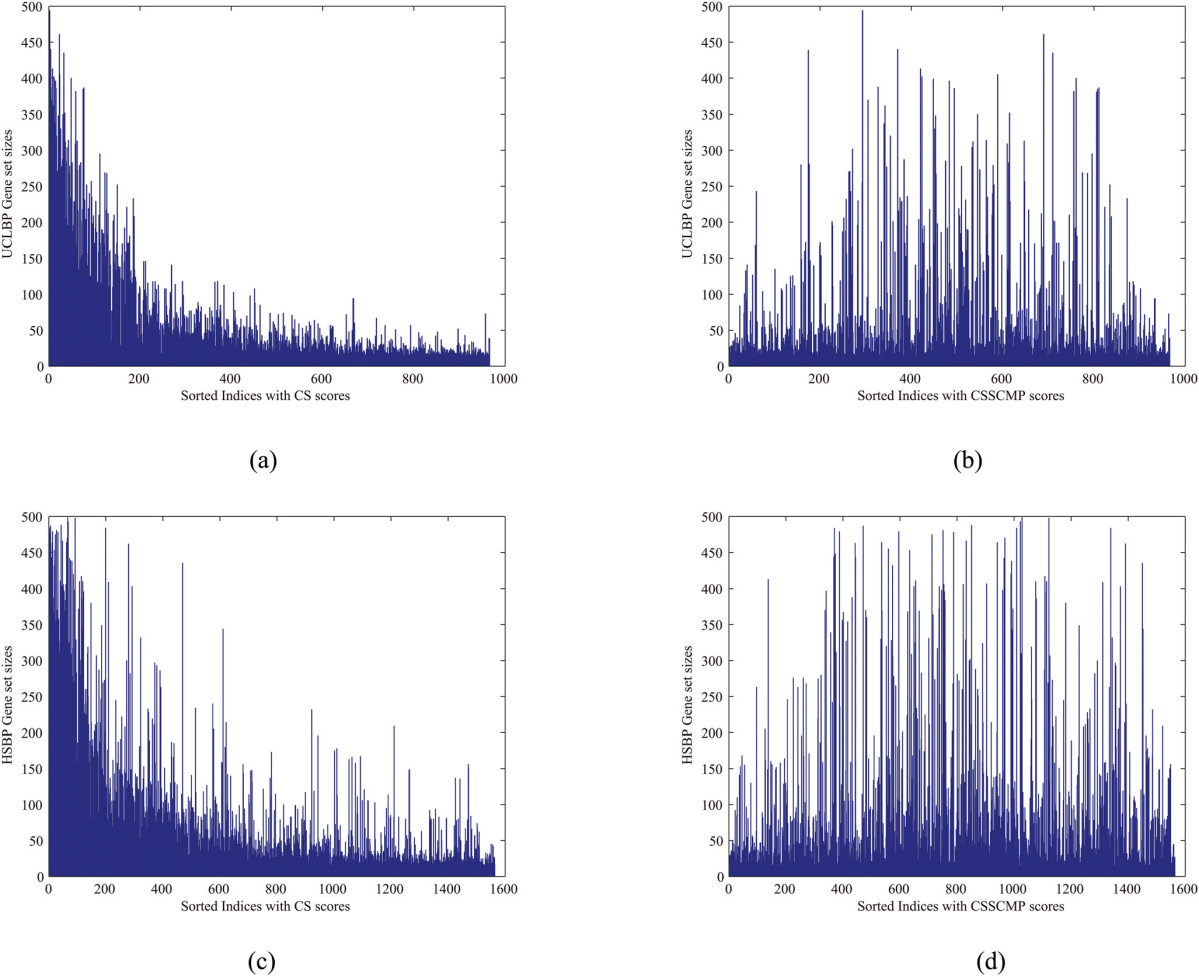
Plots of gene set sizes for two extracted gene set collections in descending order of different scores: (a)UCLBP:CS, (b)UCLBP:CSSCMP, (c)HSBP:CS, (d)HSBP:CSSCMP. The CS score heavily depends on the gene set sizes, while CSSCMP scores are insensitive to the sizes.

We next studied the CSSCMP scores with two extracted gene set collections (UCLBP and HSBP) on the hESC-CM data and on random data with different levels of variances. Specifically, we computed the CSSCMP scores with the hESC-CM data and random data with different levels of variance respectively, and computed the mean values of the top 10%, 20%, 30%, 40%, and 50% ones. These results are shown in Fig 4C and 4D, respectively. We have observed from the new experiments that both HSBP/UCLBP have much higher CSSCMP scores than those of random data. For example, the mean CSSCMP values of the top 10% gene sets are 0.22 and 0.25 for HSBP and UCLBP respectively, compared to a value of 0.03 for the random data. In general, the mean scores of random data with different levels of variance are close and significantly smaller. These results suggest that our CSSCMP score can readily separate meaningful data from random data, regardless of their variance. In addition, we ranked the gene sets in descending order of the corresponding scores, and plotted the sizes of these gene sets, as shown in Fig 5. For CS, top ranks were associated with high gene set sizes, while no such association existed for CSSCMP. These observations confirmed that CSSCMP was insensitive to the size of gene sets.

#### Gene set based consensus comparative analysis on hESC-CMs

Based on 7 datasets from 6 individual hESC-CM studies, we ordered the UCLBP and HSBP gene sets according to their CSSCMP scores to assess enrichment of gene sets in hESC-CMs relative to hESCs. For comparison, we also employed gene set enrichment analysis (GSEA) [9] on four studies with full data matrices to identify significantly enriched gene sets for each individual study [3, 5, 7].

For the HSBP gene set collection, the top 100 enriched gene sets are listed in Table 4, and details are provided in S1 and S2 Tables. Our CSSCMP method generated results largely consistent with those of GSEA. For instance, the 17 gene sets with the top CSSCMP scores were considered enriched in all four individual studies by GSEA (S1 Table). Conversely, none of the gene sets with the lowest 267 CSSCMP scores (under 0.014989) were considered enriched by GSEA in any of the four individual datasets (S2 Table). Moreover, gene sets with the largest CSSCMP scores included those known to be important for cardiac differentiation and function, e.g., ventricular-cardiac-muscle-tissue-morphogenesis (GO:0055010), myofibril-assembly (GO:0030239) and cardiac- muscle-tissue-morphogenesis (GO:0055008). This indicated that a number of gene sets were uniformly enriched in hESC-CMs relative to hESCs regardless of diverse experimental conditions, and our CSSCMP method generated results that were in accordance to those generated by GSEA and were biologically relevant.

**Table 4:** The top 100 enriched gene sets in the HSBP gene set collection identified by CSSCMP scores, with comparison to the GSEA results in four studies: study S1:purified hESC-CM (14 days) [3], S2: hESC-CM cluster (21 days) [5], S3: hESC-CM cluster (49 days) [5], and S4: purified hESC-derived ventricular (21 days) [7]. The ’1’s under the four studies mean that the corresponding gene sets are enriched, while ’0’s mean not enriched. Gene set names are represented with GO term IDs. Details can be found in supplementary files.

Gene Set Name	CSSCMP Score	PValue	S1	S2	S3	S4
GO:0055010	0.543383	8.65E-49	1	1	1	1
GO:0030239	0.489238	1.14E-38	1	1	1	1
GO:0055008	0.488207	4.81E-54	1	1	1	1
GO:0048644	0.468182	4.12E-54	1	1	1	1
GO:0060415	0.468182	5.10E-47	1	1	1	1
GO:0060047	0.456301	1.35E-29	1	1	1	1
GO:0006941	0.449198	1.63E-44	1	1	1	1
GO:0030049	0.430482	3.83E-51	1	1	1	1
GO:0033275	0.430482	2.31E-50	1	1	1	1
GO:0003208	0.421429	5.94E-48	1	1	1	1
GO:0031032	0.420956	1.74E-33	1	1	1	1
GO:0048738	0.415963	4.86E-61	1	1	1	1
GO:0055007	0.411474	6.82E-30	1	1	1	1
GO:0043462	0.391521	1.16E-25	1	1	1	1
GO:0035051	0.347174	3.50E-33	1	1	1	1
GO:0030048	0.338497	5.50E-39	1	1	1	1
GO:0055002	0.327973	3.91E-29	1	1	1	1
GO:0048146	0.320188	4.09E-19	0	0	0	1
GO:0060411	0.304546	8.66E-12	0	1	0	0
GO:0008016	0.304546	6.78E-31	1	1	1	1
GO:0060537	0.294946	1.36E-52	1	1	1	1
GO:0003281	0.290107	1.07E-11	1	0	0	1
GO:0055001	0.28667	3.03E-25	1	1	1	1
GO:0043588	0.285295	1.02E-10	0	1	1	0
GO:0003279	0.271798	1.26E-13	0	1	0	1
GO:0010574	0.269051	1.95E-10	1	1	1	1
GO:0048747	0.266043	2.92E-11	1	1	1	1
GO:0003007	0.261514	7.47E-37	1	1	1	1
GO:0032835	0.26123	2.16E-15	1	1	0	1
GO:0030198	0.260027	9.77E-22	1	1	1	1
GO:0043062	0.260027	3.02E-21	1	1	1	1
GO:0006942	0.258222	2.36E-10	0	1	1	1
GO:0007517	0.25773	3.50E-48	1	1	1	1
GO:0010718	0.257178	9.58E-11	0	1	1	1
GO:0006937	0.256946	4.92E-20	0	1	1	1
GO:0048546	0.254719	5.10E-09	0	0	0	0
GO:0045669	0.252507	1.00E-11	0	1	0	1
GO:2000379	0.251003	1.16E-10	0	0	0	1
GO:0006936	0.245563	4.83E-57	1	1	1	1
GO:0034446	0.237467	2.30E-07	0	0	0	0
GO:0045778	0.236426	7.10E-12	1	1	1	1
GO:0042698	0.233486	1.01E-07	1	0	0	1
GO:0048742	0.233316	1.12E-10	1	1	0	1
GO:0071277	0.229239	2.53E-08	0	0	0	1
GO:0001937	0.228681	1.93E-08	1	1	1	1
GO:0003012	0.228532	9.85E-50	1	1	1	1
GO:0061061	0.220708	8.53E-52	1	1	1	1
GO:0010717	0.220547	4.80E-11	0	1	0	1
GO:0051153	0.21591	2.33E-12	1	1	1	1
GO:0014910	0.214376	1.18E-06	0	1	1	1
GO:0051384	0.212651	5.75E-06	0	1	1	0
GO:0003143	0.212161	3.24E-08	1	1	1	1
GO:0030501	0.210805	7.57E-08	1	1	0	1
GO:0048566	0.209894	1.37E-06	0	0	0	0
GO:0048010	0.209683	1.72E-06	0	1	1	1
GO:0051592	0.209566	1.67E-14	0	1	1	1
GO:0007507	0.20728	4.53E-40	1	1	1	1
GO:0048706	0.205883	2.00E-09	0	1	1	0
GO:0048565	0.204852	1.39E-11	0	0	0	1
GO:0010595	0.202956	4.50E-11	0	0	0	1
GO:0048705	0.199867	5.69E-11	0	0	0	0
GO:0048145	0.197553	3.36E-11	0	0	0	1
GO:0042692	0.195463	7.82E-24	1	1	1	1
GO:0035050	0.194776	1.33E-07	0	1	0	1
GO:0010594	0.190132	3.85E-13	0	0	0	1
GO:0045884	0.190091	8.39E-05	0	1	0	0
GO:0071407	0.190091	6.78E-05	1	1	1	1
GO:0031960	0.18984	2.93E-05	0	1	1	0
GO:0060349	0.188785	3.62E-06	0	0	0	0
GO:0016202	0.188694	1.14E-10	0	1	0	1
GO:0001947	0.187835	3.93E-06	0	1	0	1
GO:0002576	0.185384	1.10E-19	1	1	1	1
GO:0003158	0.181819	3.36E-05	1	0	0	1
GO:0060485	0.181185	1.49E-12	0	0	0	1
GO:0002062	0.1801	1.28E-05	0	1	0	1
GO:0008015	0.18006	5.05E-25	1	1	1	1
GO:0030324	0.178811	2.94E-06	1	0	0	1
GO:0051279	0.178811	1.28E-04	0	0	0	1
GO:0071560	0.177386	6.37E-05	1	1	1	1
GO:0050880	0.177006	3.62E-04	0	0	0	1
GO:0010596	0.175803	1.53E-04	0	0	1	1
GO:0003073	0.175427	5.00E-07	1	1	1	0
GO:0001837	0.175134	4.00E-06	0	0	0	1
GO:0050680	0.174395	9.11E-10	0	0	0	1
GO:0030500	0.173797	5.93E-08	0	1	0	1
GO:0030323	0.173521	3.15E-06	1	0	0	1
GO:0051216	0.173308	5.39E-09	0	0	0	1
GO:0001936	0.173129	1.18E-10	0	1	1	1
GO:0061371	0.172795	2.47E-05	0	1	0	1
GO:0060348	0.170818	4.62E-07	0	0	0	0
GO:2000377	0.170762	4.32E-07	0	0	0	0
GO:0003151	0.169787	1.94E-05	0	0	0	0
GO:0001649	0.16738	4.68E-06	0	0	0	1
GO:0009887	0.166493	3.73E-40	1	1	1	1
GO:0050729	0.165622	1.88E-05	1	0	1	1
GO:0009187	0.165385	2.98E-08	0	1	1	1
GO:0030326	0.164233	4.52E-05	0	0	0	1
GO:0060688	0.164087	5.10E-04	0	0	0	0
GO:0006939	0.163509	2.56E-04	0	0	0	0
GO:0006970	0.163019	1.87E-03	0	0	0	0

In addition, CSSCMP identified enrichment of potentially important gene sets that were not detected by GSEA based on individual studies. Examples included positive-regulation-of-reactive-oxygen-species-metabolic-process (GO:2000379) and response-to-glucocortic-stimulus (GO:0051384) etc., as shown in Tables 5 and 6. Reactive oxygen species is important for cardiac differentiation of hESCs [30] but surprisingly it was not considered enriched by GSEA in any of the individual studies. Response-to-glucocorticoid- stimulus (GO:0051384) was enriched in two out of four datasets, but its role in hESC cardiac differentiation is unclear and requires further attention. Examination of genes associated with glucocorticoid-stimulus showed that ADAM9, AQP1, GOT1, ISL1, SLIT2 and SLIT3 were significantly upregulated in more than 4 of the 7 datasets and may be responsible for mediating the effect of this stimulus. Moreover, false positive results can arise from individual studies partly as a reflection of the specific conditions used in the experiment, and may not represent the biological entities examined. For instance, GSEA examination of the Cao et al. data set showed that genes involved in complement-activation (GO:0006956) was very significantly enriched in hESC-CMs. However, CSSCMP of four datasets showed that this gene set was only enriched in this single study, with a CSSCMP rank of 1349 and score of 0.012251, which is non-significant. By considering multiple datasets, false positives related to specific biological conditions may be reduced. More detailed results of different ranking between CSSCMP and results on GSEA with individual studies are listed in S3 Table.

**Table 5:** Selected gene sets enriched in hESC-CMs relative to hESCs. CSSCMP score and PVal were calculated based on seven data sets. For comparison, GSEA results in four studies are shown: study S1:purified hESC-CM (14 days) [3], S2: hESC-CM cluster (21 days) [5], S3: hESC-CM cluster (49 days) [5], and S4: purified hESC-derived ventricular (21 days) [7]. The ’1’s under the four studies mean that the corresponding gene sets are enriched, while ’0’s mean not enriched. Details can be found in the supplementary files.

Gene Set Name	Score	PVal	S1	S2	S3	S4
response-to-glucocorticoid-stimulus	0.21	5.7e-006	0	1	1	0
positive-regulation-of-reactive-oxygen-species-metabolic-process	0.25	1.2e-010	0	0	0	1

**Table 6:** Genes affiliated with “response-to-glucocorticoid-stimulus”. Fold changes in all seven data sets are also shown. S1-4 are defined as in (A). S5:hESC-CM cluster (12 days) [1], S6:hESC-CM cluster (within 22 days) [2], S7:purified hESC-CM (22 days) [4]. Non-significant changes are shown as ’0’.

TargetID	S1	S2	S3	S4	S5	S6	S7
ADAM9	4.1	3.6	5.9	0	3.6	0	0
AQP1	12.0	11.4	14.2	2.7	10	42	0
CALCR	0	0	0	0	0	0	0
GHRHR	0	0	0	0	0	0	0
GOT1	0	2.7	3.8	3	0	0	2.8
IL10	0	0	0	0	0	0	0
IL1RN	0	0	0	0	0	0	0
IL6	0	0	2.4	3.9	0	0	0
ISL1	8.6	2.7	0	2.8	7.8	43	0
NR3C1	0	0	0	0	0	0	39.3
PDCD7	0	0	0	0	0	0	0
REST	0	0	0	0	0	0	0
SLIT2	21.3	6.2	9	2.8	0	0	0
SLIT3	16.1	11.4	13	33.5	12.6	39	0
TNF	0	0	0	2.2	0	0	0
UBE2L3	0	0	0	0	0	0	0

**Fig 6:**
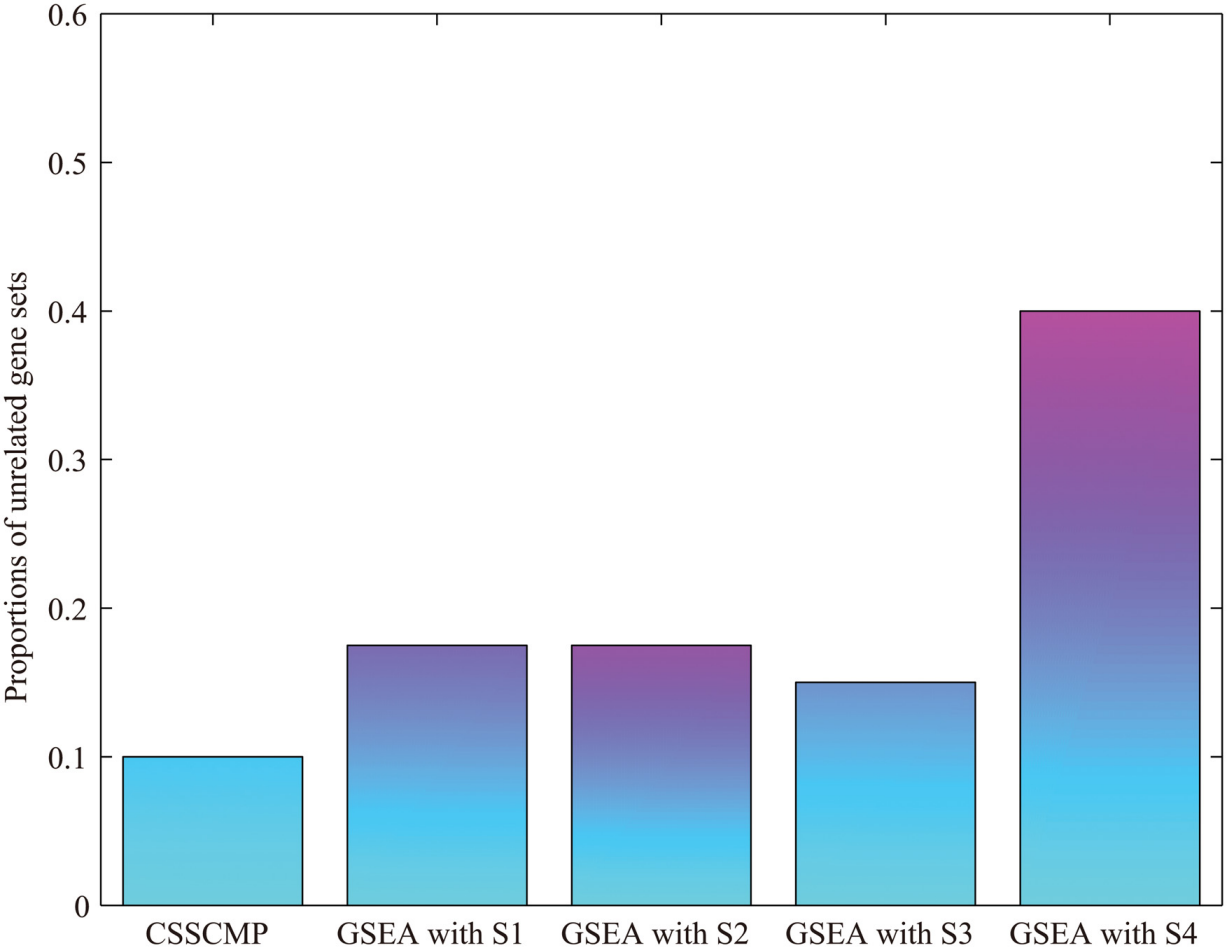
Proportion of unrelated HSBP gene sets identified by CSSCMP and GSEA with individual studies: S1:purified hESC-CM (14 days) [3], S2: hESC-CM cluster (21 days) [5], S3: hESC-CM cluster (49 days) [5], and S4: purified hESC-derived ventricular (21 days) [7]. CSSCMP identified the smallest proportion of unrelated gene sets.

To further compare the performance of CSSCMP and GSEA, we also identified unrelated gene sets that bear no obvious relationship to heart development and function (e.g. skin development, platelet degranulation) among the top gene sets identified by CSSCMP with those identified by GSEA of four individual studies (see S4 Table). Fig 6 shows the number of unrelated gene sets among the top 40 ones in each study. CSSCMP identified the smallest number of unrelated gene sets compared to GSEA of the individual studies. Importantly, these unrelated gene sets identified by GSEA reflect the purity and biological properties of the samples used in the individual studies. The samples used in Cao et al. [3] (besides Poon et al. [7]) have the highest purity and this study has a smaller number of unrelated gene sets than Jane et al. [5]. Poon et al. [7] used lentiviral selection to isolate hESC-VCMs, and consequently have a large number of gene sets related to inflammation, such as positive-regulation-of-cytokine-production(GO:0001819), among its top 40 gene sets. In conclusion, these show that our CSSCMP is superior in its ability to avoid false positive gene sets and are less sensitive to sample heterogeneity.

**Fig 7:**
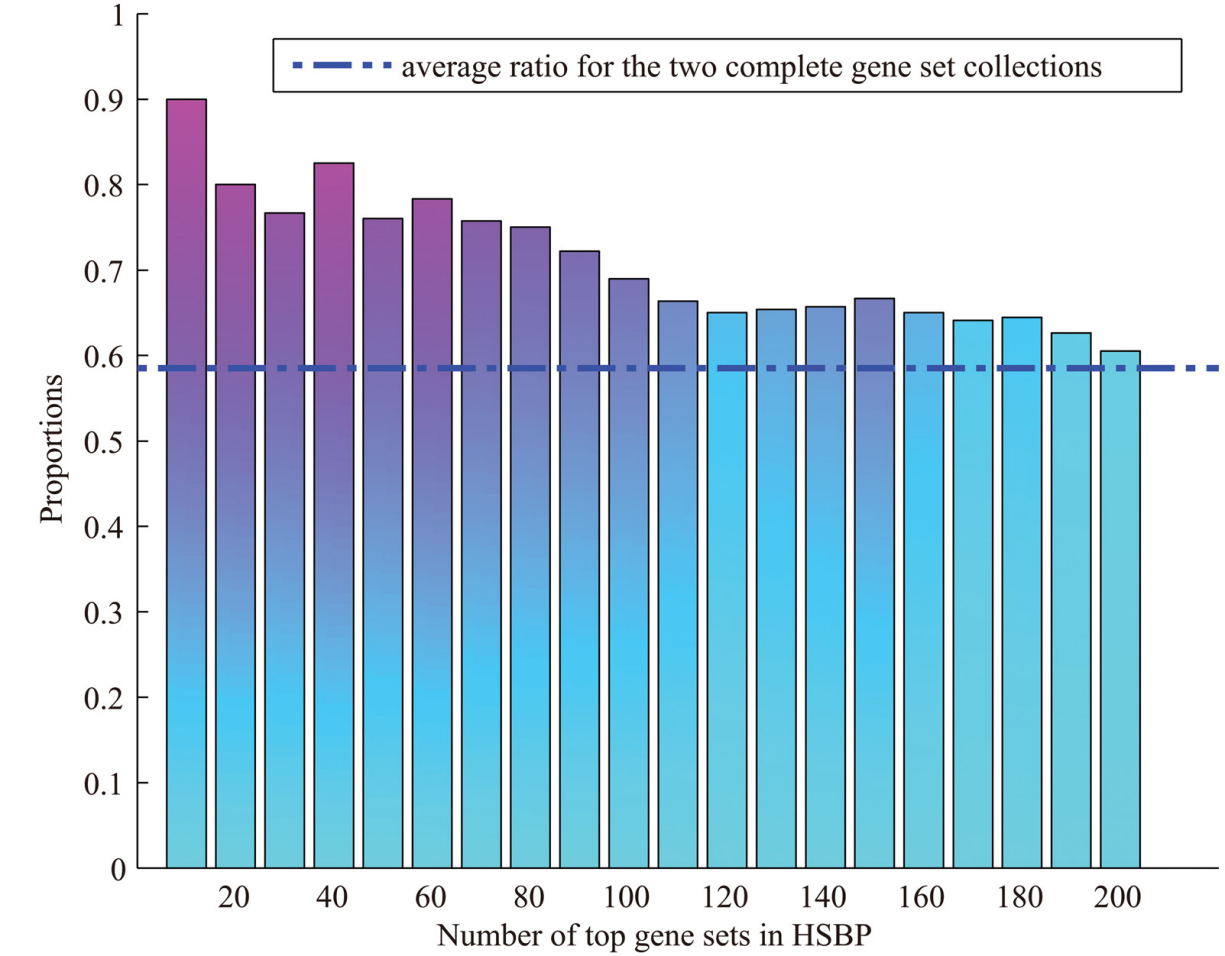
Proportion of common top gene sets in both gene set collections with respect to those in HSBP. The average ratio for the two complete gene set collections (915/1564) is plotted as a horizontal line.

For the UCLBP gene set collection, the top 100 sets are listed in Table 7, and details are provided in S5 Table. Scores of the members of the whole gene set list are provided in S6 Table. Analysis with the UCLBP generated similar results as the HSBP gene set collection. As shown in Fig 7, a large proportion of the top gene sets were the same in both gene set collections, and this proportion was significantly larger than the average ratio for the two complete gene set collections (i.e., 915/1564, plotted as a horizontal line). Specifically, 9 of the top 10 gene sets for two gene set collections were the same. This showed that most of the enriched gene sets of HSBP came from UCLBP, which is highly related to biological processes associated with heart development.

**Table 7:** The top 100 enriched gene sets in the UCLBP gene set collection identified by CSSCMP scores, with comparison to the GSEA results in four studies: study S1:purified hESC-CM (14 days) [3], S2: hESC-CM cluster (21 days) [5], S3: hESC-CM cluster (49 days) [5], and S4: purified hESC-derived ventricular (21 days) [7]. The ’1’s under the four studies mean that the corresponding gene sets are enriched, while ’0’s mean not enriched. Gene set names are represented with GO term IDs. Details can be found in supplementary files.

Gene Set Name	CSSCMP Score	PValue	S1	S2	S3	S4
GO:0055010	0.537743	7.50E-28	1	1	1	1
GO:0055008	0.48029	1.85E-27	1	1	1	1
GO:0030239	0.47623	6.75E-19	1	1	1	1
GO:0031032	0.47623	1.51E-17	1	1	1	1
GO:0048644	0.459429	2.37E-28	1	1	1	1
GO:0060415	0.459429	9.70E-25	1	1	1	1
GO:0060047	0.449585	1.66E-15	1	1	1	1
GO:0006941	0.442395	4.89E-24	1	1	1	1
GO:0030049	0.429944	2.57E-27	1	1	1	1
GO:0033275	0.429944	2.64E-28	1	1	1	1
GO:0003208	0.424462	2.70E-23	1	1	1	1
GO:0055007	0.417154	6.84E-17	1	1	1	1
GO:0048738	0.412149	7.38E-31	1	1	1	1
GO:0030048	0.398017	4.71E-24	1	1	1	1
GO:0035051	0.35252	1.30E-16	1	1	1	1
GO:0032835	0.350363	8.60E-10	1	1	1	1
GO:0008016	0.340415	1.35E-19	1	1	1	1
GO:0048146	0.328322	2.83E-10	1	0	0	1
GO:0030198	0.314836	1.50E-12	1	1	1	1
GO:0043062	0.314836	7.10E-14	1	1	1	1
GO:0051592	0.298392	3.85E-11	0	1	1	1
GO:0060411	0.295956	4.71E-06	0	1	0	0
GO:0055001	0.290475	1.63E-11	1	1	1	1
GO:0060537	0.28917	8.08E-24	1	1	1	1
GO:0045669	0.278294	1.92E-06	0	1	1	1
GO:0048747	0.278294	2.18E-05	1	1	1	0
GO:2000379	0.271188	4.41E-06	0	0	0	1
GO:0045778	0.267497	1.57E-06	1	1	1	1
GO:0048742	0.267235	2.25E-06	1	1	1	1
GO:0006942	0.263389	1.16E-05	1	1	1	1
GO:0003007	0.260764	1.82E-17	1	1	1	1
GO:0006937	0.260726	1.21E-10	0	1	1	1
GO:0051153	0.260023	1.19E-07	1	1	1	1
GO:0003279	0.259192	2.01E-06	1	1	0	1
GO:0042698	0.254542	2.62E-04	1	1	0	1
GO:0007517	0.253239	6.71E-24	1	1	1	1
GO:0048706	0.250887	5.46E-05	0	1	1	1
GO:0006936	0.245804	1.19E-27	1	1	1	1
GO:0030029	0.236588	1.63E-16	1	1	1	1
GO:0048145	0.233876	2.57E-06	0	0	0	1
GO:0061061	0.231061	3.45E-25	1	1	1	1
GO:0030501	0.230693	1.91E-04	1	1	1	1
GO:0031589	0.22844	7.70E-07	1	1	1	1
GO:0030855	0.226668	5.74E-08	1	1	1	1
GO:0016202	0.224865	1.75E-06	0	1	1	1
GO:0003143	0.223481	1.10E-04	1	1	1	1
GO:0048705	0.222536	9.56E-06	0	0	0	0
GO:0002576	0.219484	1.70E-10	1	1	1	1
GO:0008544	0.218811	2.53E-07	1	1	1	1
GO:0051384	0.217999	1.79E-03	0	1	1	0
GO:0042692	0.21723	4.88E-12	1	1	1	1
GO:2000377	0.215751	9.23E-05	0	0	0	0
GO:0007507	0.21305	8.17E-18	1	1	1	1
GO:0048565	0.212823	3.06E-04	0	0	0	1
GO:0014910	0.212061	2.74E-03	0	1	1	1
GO:0035050	0.211023	3.76E-04	1	1	1	1
GO:0007160	0.208255	1.13E-04	1	1	1	1
GO:0009887	0.20782	2.23E-20	1	1	1	1
GO:0009888	0.202841	2.64E-28	1	1	1	1
GO:0010717	0.202718	7.85E-04	0	0	0	1
GO:0030326	0.201556	1.91E-03	0	0	0	1
GO:0010595	0.200789	8.13E-05	0	0	0	1
GO:0003073	0.198885	1.09E-03	1	1	1	1
GO:0001947	0.196074	2.69E-03	1	1	0	1
GO:0030324	0.194044	4.45E-03	1	0	0	0
GO:0003158	0.19379	1.09E-02	1	0	0	1
GO:0060485	0.192594	1.92E-05	0	1	0	1
GO:0051216	0.190452	9.34E-04	0	0	0	1
GO:0048010	0.188766	9.72E-03	0	1	1	1
GO:0060393	0.188766	8.48E-03	0	0	0	1
GO:0043009	0.185924	8.15E-06	0	1	1	1
GO:0030500	0.185634	1.53E-03	1	1	1	1
GO:0071241	0.185416	2.74E-03	0	1	1	0
GO:0030323	0.184535	7.77E-03	1	0	0	0
GO:0030036	0.184286	8.25E-07	1	1	1	1
GO:0008015	0.183776	1.40E-09	1	1	1	1
GO:0021537	0.183023	8.38E-04	1	1	1	1
GO:0006887	0.182786	1.68E-07	1	1	1	0
GO:0072073	0.182371	2.58E-02	0	0	0	0
GO:0001822	0.181863	7.88E-06	1	1	1	1
GO:0050777	0.181027	1.07E-02	1	1	1	1
GO:0007179	0.179464	8.63E-04	0	1	0	1
GO:0010594	0.178634	1.27E-04	0	0	0	1
GO:0003151	0.177803	7.45E-03	0	0	0	0
GO:0048592	0.177803	6.83E-03	1	1	1	0
GO:0061371	0.176933	8.52E-03	0	1	0	1
GO:0045667	0.175193	1.11E-03	0	1	0	1
GO:0042476	0.174149	1.26E-02	0	1	1	0
GO:0048598	0.173476	1.13E-06	0	0	0	1
GO:0060541	0.173453	1.25E-02	1	0	0	0
GO:0050680	0.171278	7.10E-03	0	0	0	1
GO:0000079	0.170359	7.84E-03	1	1	1	1
GO:0072001	0.170243	2.50E-05	1	1	1	1
GO:0035303	0.169498	1.11E-02	0	1	0	1
GO:0021953	0.169277	4.56E-02	0	1	1	0
GO:0030278	0.169006	1.52E-04	1	1	0	1
GO:0001654	0.168668	7.37E-04	1	1	0	1
GO:0048646	0.168465	1.11E-10	1	1	1	1
GO:0051924	0.167965	1.07E-04	1	1	1	1
GO:0048762	0.167885	2.07E-03	0	1	0	1

## Discussion

Application of hESC-CM for drug discovery and transplantation requires a thorough molecular characterization of these cells, but this is compromised by variations in experimental conditions among different studies. Conventional methods are unsuitable for consensus analysis of hESC-CM microarray data. To bridge this gap, we propose a new consensus comparison analysis approach, CSSCMP, and identified novel changes in genes and gene sets that occur in hESC-CM irrespective of different experimental variables. Based on the consensus information of different individual studies, our proposed CSSCMP approach has a number of advantages: (1) detection of randomness in the input; (2) improvement of efficiency; (3) mitigation of the problem of gene set size dependence; and (4) integration of information from multiple heterogeneous data sources.

The current study points to a number of important future research directions from both computational and biological perspectives. From a computational perspective, an interesting improvement of the current approach is to replace the current binary matrix entries with real values for each individual study. From a biological perspective, a potential extension of the present work is to study the interaction between the identified gene sets and microRNAs.

## Supporting Information

S1 Table Top 100 enriched gene sets based on CSSCMP scores on HSBP gene set collection. (XLS)

S2 Table Enriched gene sets based on CSSCMP scores on HSBP gene set collection. (XLS)

S3 Table Detailed results of different ranking between CSSCMP and results on GSEA with individual studies. (XLS)

S4 Table Top 40 enriched gene sets identified by CSSCMP and GSEA in individual studies. Unrelated gene sets are highlighted in red.
(XLS)

S5 Table Top 100 enriched gene sets based on CSSCMP scores on UCLBP gene set collection. (XLS)

S6 Table Enriched gene sets based on CSSCMP scores on UCLBP gene set collection. (XLS)

S1 Data Data and implementation codes in Matlab. (ZIP)
